# Sensing Single-Molecule Magnets with Nitrogen-Vacancy Centers

**DOI:** 10.1021/acs.nanolett.5c05066

**Published:** 2026-01-29

**Authors:** Ariel Smooha, Jitender Kumar, Dan Yudilevich, John W. Rosenberg, Valentin Bayer, Rainer Stöhr, Andrej Denisenko, Tatyana Bendikov, Anna Kossoy, Iddo Pinkas, Hengxin Tan, Binghai Yan, Biprajit Sarkar, Joris van Slageren, Amit Finkler

**Affiliations:** § Department of Chemical and Biological Physics, 34976 Weizmann Institute of Science, 7610001 Rehovot, Israel; ⊥ Institute of Physical Chemistry, 9149 University of Stuttgart, Pfaffenwaldring 55, 70569 Stuttgart, Germany; ∥ Third Institute of Physics, IQST and ZAQuant, University of Stuttgart, 70569 Stuttgart, Germany; ¶ Department of Chemical Research Support, Weizmann Institute of Science, 7610001 Rehovot, Israel; # Department of Condensed Matter Physics, Weizmann Institute of Science, 7610001 Rehovot, Israel; ∇ Institute of Inorganic Chemistry, University of Stuttgart, Pfaffenwaldring 55, 70569 Stuttgart, Germany; ○ Institut für Chemie und Biochemie, 9166 Freie Universität Berlin, 14195 Berlin, Germany

**Keywords:** nitrogen-vacancy centers, single-molecule magnets, quantum sensing, spin relaxometry, noise spectrum density

## Abstract

Single-molecule magnets (SMMs) are molecules that can function as nanoscale magnets with potential use as magnetic memory bits. While SMMs can retain magnetization at low temperatures, characterizing them on surfaces and at room temperature remains challenging and requires specialized nanoscale techniques. Here, we use single nitrogen-vacancy (NV) centers in diamond as a highly sensitive, broadband magnetic field sensor to detect the magnetic noise of cobalt-based SMMs deposited on a diamond surface. We measured the NV relaxation and decoherence times at 296 K and at 5–8 K, observing a significant influence of the SMMs on them. From this, we infer the SMMs’ magnetic noise spectral density (NSD) and underlying magnetic properties. Moreover, we observe the effect of an applied magnetic field on the SMMs’ NSD at low temperatures. The method provides nanoscale sensitivity for characterizing SMMs under realistic conditions relevant to their use as surface-bound memory units.

## Graphical abstract

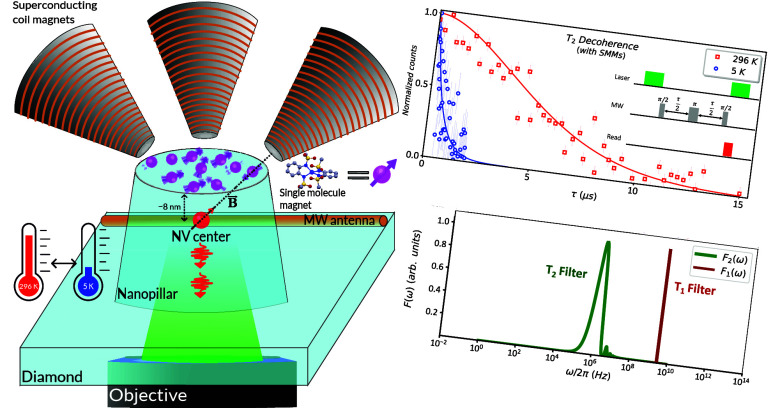

Single-molecule magnets (SMMs) are molecules that can behave as individual nanomagnets. SMMs are promising candidates for magnetic data storage with ultrahigh data densities due to their nanometer size. They consist of an inner core of one or more metal ions with a surrounding shell of organic ligands^1^ that can be tailored to bind them on surfaces.^2,3^ Due to their mesoscopic size, they can be used to study the transition from the classical to the quantum mechanical regime, e.g., effects such as quantum tunneling of magnetization at low temperatures (LTs).^4^

Since the discovery of SMMs in 1993,^5,6^ these materials have attracted considerable interest for their potential applications in quantum computing^7,8^ and spintronics.^2,9^ However, a major challenge of utilizing SMMs for such applications is their fast spin dynamics at elevated temperatures due to stochastic magnetic fluctuations. One of the key parameters to characterize SMMs is their blocking temperature, *T*_B_. Below this temperature, the magnetic moment of the molecule will be thermally stable (or “blocked”), and at higher temperatures, it behaves like a superparamagnet, where the thermal fluctuations dominate, such that the average magnetization will be zero in the absence of an external magnetic field.

Indeed, over the last three decades, there has been a constant effort to increase the blocking temperature of SMMs.^10^ Here, another important aspect is the challenge in their detection and characterization at the nanoscale. Conventional methods for SMMs’ characterization include superconducting quantum interference device magnetometry,^6,11^ electron spin resonance,^12^ inelastic neutron scattering,^13^ and X-ray spectroscopy.^14^ Other techniques include Mössbauer spectroscopy^15^ and magnetic force microscopy.^16^ However, these techniques typically require either a macroscopic amount of material, LTs, or an ultrahigh vacuum and have a limited detection bandwidth for magnetic fluctuations. Furthermore, these techniques are less optimal for studying SMMs deposited on a surface, a geometry where they can practically function as memory units.

Here we demonstrate that it is possible to sense SMMs at nanoscale volumes using a quantum sensor in the form of a single nitrogen-vacancy (NV) center in diamond, which allows us to measure the spectral density of magnetic noise of these molecules when applied on the diamond’s surface. By comparing the nanoscale measurements to bulk ones,^17^ we show below the differences observed between them and provide a path to further exploration of other SMMs.

The NV center covers 10 orders of magnitude of frequency bandwidth, ranging from subhertz up to the gigahertz regime, and functions at a broad range of temperatures.^18^ Moreover, the NV center is capable of sensing small magnetic moments outside of the diamond crystal, down to a single electron spin.^19–21^ It consists of a substitutional nitrogen and an adjacent vacancy, with a nanoscopic detection volume.^22^ In its negatively charged state, it is a spin-1 system with spin-dependent photoluminescence, enabling optical detection of magnetic fields.^23,24^

## NV-Based Relaxation Measurements

To investigate the magnetic noise generated by surface-deposited SMMs, we employ both *T*_1_ longitudinal relaxometry and *T*_2_ decoherence using shallow NV centers in diamond (8 ± 3 nm from the surface; Figure 1). Although the mean magnetic field ⟨*B*⟩ produced by an SMM spin bath may average to zero, magnetic field fluctuations generate a nonzero root-mean-square field $\sqrt{\langle B^{2}}\rangle$ with a random phase. Such stochastic magnetic fields are inherently challenging to detect on the nanoscale. However, NV centers provide a powerful avenue for sensing them by monitoring their quantum-spin relaxation dynamics.^25,26^ After initialization of the NV center into a well-defined spin state, interactions with its surrounding environment lead to spin relaxation. The relaxation is induced by intrinsic components from spin impurities and the vibrational lattice dynamics and extrinsic components from the environment, such as nearby spin systems, to which the NV can be deliberately exposed. Relaxation in spin systems may occur through two primary channels: decoherence *T*_2_, which is sensitive to low-frequency fluctuations (kilohertz to megahertz), and longitudinal relaxation *T*_1_, which is sensitive to noise at a frequency near the NV center Larmor frequency (∼2.87 GHz at low fields).

**Figure 1:**
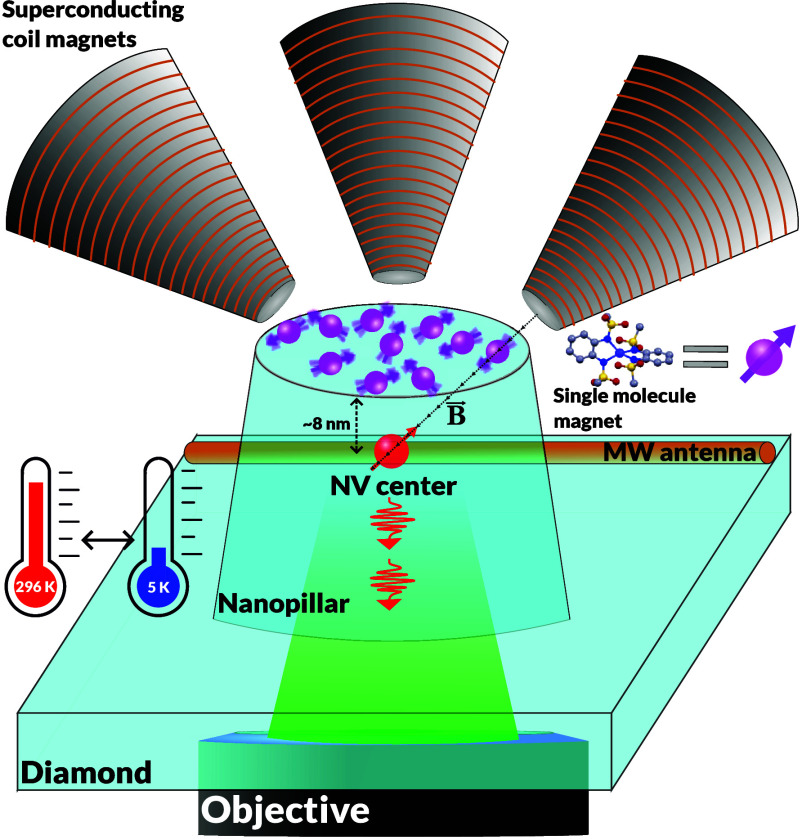
General scheme of the LT NV setup (not to scale). SMMs are deposited on top of a diamond nanopillar (∼450 nm diameter). A confocal microscopy setup is used to excite the NV center with a 520 nm laser, and emitted photons are collected and measured. A direct-current magnetic field is applied with three superconducting coils. The LT system can reach 5 K and resides in ultrahigh-vacuum conditions. A MW antenna is used for spin-state manipulation.

## Sensing SMMs

To sense cobalt-based SMMs, we deposited them onto a diamond membrane hosting shallow NV centers (see Section S8 for a detailed description). The full chemical formula of the SMM tetrahedral complex is (HNEt_3_)_2_[Co^II^(L^2–^)^2^] where the ligand L stands for 1,2-bis­(methanesulfonamido)­benzene.^17^ Based on a 1 mM solution (in acetonitrile) and a spherical-cap geometry, the NV is expected to sense ∼240 molecules, within an effective sensing radius of 20 nm obtained from a simulation [sensing volume of ∼(20 nm)^3^; see Section S1]. Similar NV sensing ranges were also experimentally found when sensing transition metals.^27^

We thoroughly characterized the deposited cobalt-based SMM layer on diamond with three spectroscopic techniques. Specifically, X-ray diffraction and Raman spectroscopy indicate that the deposited layer retains the SMM molecular structure, and X-ray photoelectron spectroscopy (XPS) demonstrated that the cobalt is found in the Co^2+^ oxidation state (see Sections S2 and S10). A confocal microscopy setup was used to optically polarize and read out individual NV centers. We first carried out *T*_2_ coherence time measurements at room temperature (RT, ∼296 K) and low temperature (LT, ∼5 K) in the presence of SMMs. Measurements were repeated across multiple NV centers to ensure reproducibility and to account for variations in local environments. In a *T*_2_ measurement, we utilize the spin-echo pulse sequence for tracking the decay profile of the superposition state $\left|\psi \right\rangle =\frac{1}{\sqrt{2}}\left(\left|0\right\rangle +\left|1\right\rangle \right)$ with time. A representative *T*_2_ measurement is shown in Figure 2. In the presence of SMMs, we observed a significant reduction by approximately 1 order of magnitude in the coherence time *T*_2_ of the NV center when cooling from 296 K (RT) to 5 K (LT), under a low magnetic field (*B*_0_ ∼ 20 G).

**Figure 2:**
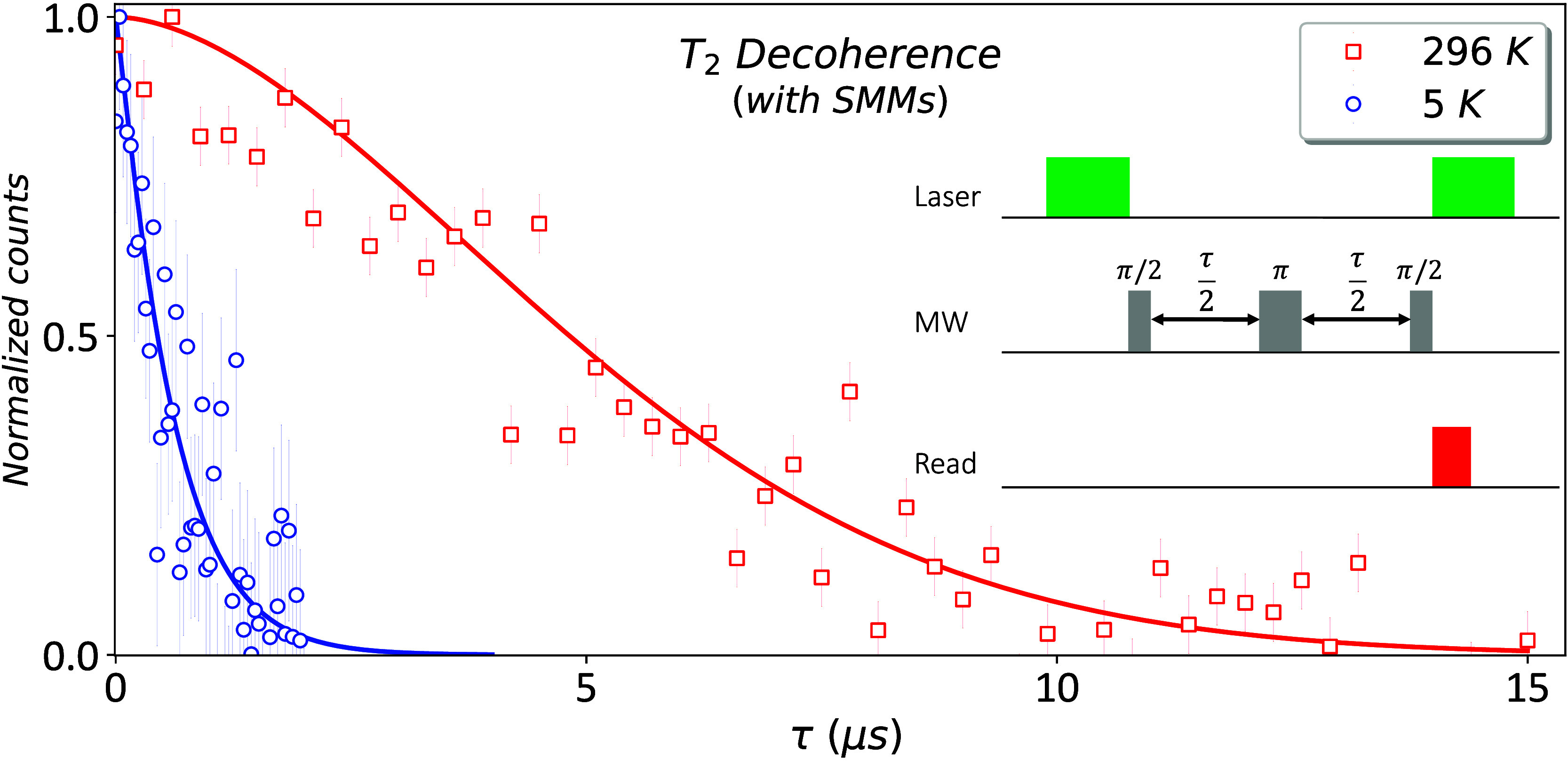
Effect of the cobalt-based SMMs at RT and 5 K. The NV center (NV17) *T*_2_ coherence curve, in the presence of SMMs, at 296 K (red) and 5 K (blue), with *T*_2_ = 5.9 ± 0.3 μs and *T*_2_ = 0.62 ± 0.09 μs, respectively. The data were fitted based on Section S3. Inset: *T*_2_ pulse sequence.

We also performed *T*_1_ measurements at RT and 5 K by optically initializing the NV center to the |0⟩ state and tracking the relaxation profile of the system without microwave (MW) radiation.^28^ While the *T*_2_ measurements exhibited a clear and pronounced temperature dependence, the *T*_1_ measurements showed only a modest increase in relaxation time at 5 K compared to RT (Figure S4).

To confirm that the observed effects at 5 K were induced by the SMMs and not by other possible sources of magnetic noise, such as surface or lattice spin species, we conducted comparative *T*_2_ measurements at 5 K after removing the SMMs from the diamond surface via a triacid-cleaning protocol.^29^ A representative postcleaning *T*_2_ measurement is presented in Figure 3a. In the absence of SMMs, we observed a substantial recovery of the *T*_2_ coherence times at 5 K, with values increasing by up to a factor of 5 compared to those measured in the presence of SMMs. We also conducted comparative *T*_1_ measurements at 5 K after removing the SMMs. In this case, we observed a pronounced reduction in the *T*_1_ relaxation time of the NV centers at 5 K in the presence of SMMs, amounting to approximately 1–2 orders of magnitude relative to the cleaned sample (Figure 3b). These findings suggest that the enhanced relaxation rate observed at 5 K arises from magnetic field fluctuations originating from the SMMs. The overall trend is consistent across all measured NVs (see Section S4).

**Figure 3:**
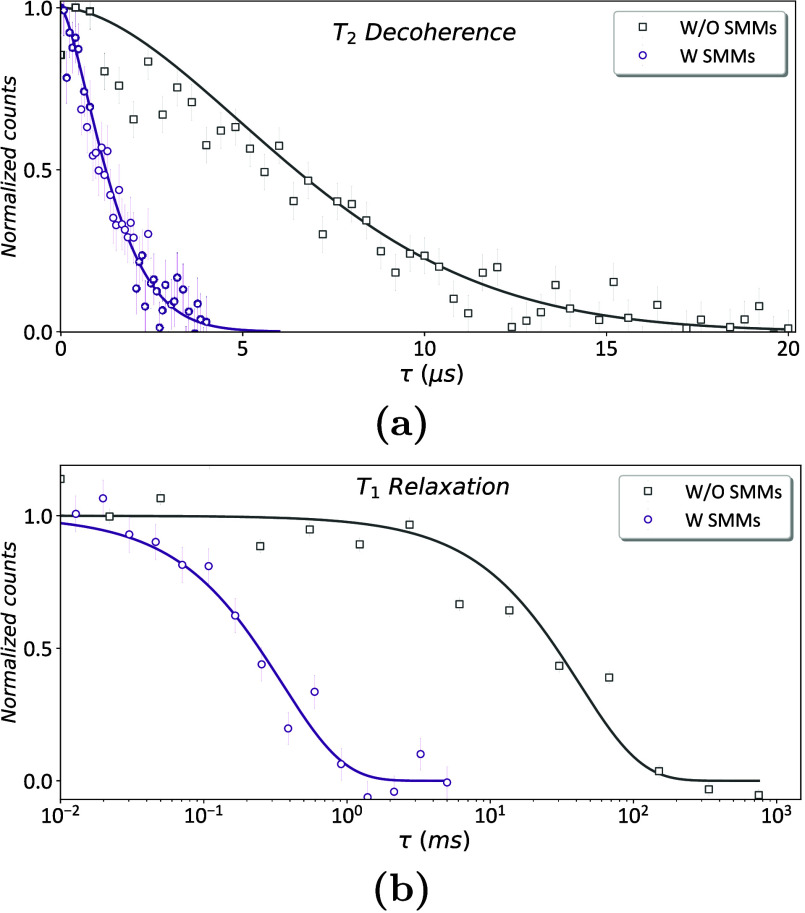
Effect of the cobalt-based SMMs at ∼5 K. (a) NV center (NV24) *T*_2_ coherence curves in the presence (purple) and absence (gray) of SMMs, with *T*_2_ = 0.80 ± 0.04 μs and *T*_2_ = 4.0 ± 0.2 μs, respectively. (b) *T*_1_ relaxation curves (NV24) in the presence (purple) and absence (gray) of SMMs with *T*_1_ = 0.35 ± 0.05 ms and *T*_1_ = 22 ± 4 ms, respectively. The data were fitted based on Section S3.

The observed trend can be explained based on a general model for electronic spin noise, describing the behavior of magnetic fluctuators. In this model, the dynamics of the magnetic spin noise can be described as an Ornstein–Uhlenbeck process,^30,31^ which is a stationary Gauss–Markov process, with the following autocorrelation function:

1 $$A\left(t\right)=\left\langle B\left(0\right)\;B\left(t\right)\right\rangle =\left\langle B^{2}\right\rangle \;\exp\left(-\frac{t}{\tau_{c}}\right)$$

where *B*(*t*) is the time-dependent magnetic field induced by the noise bath and τ_c_ is the correlation time, which is the “memory time” of the environmental noise. The Fourier transform of eq 1 yields the normalized magnetic noise spectrum density (NSD)^25^

2 $$S\left(\omega ,T,E_{a}\right)=\frac{2}{\pi }\frac{\tau_{c}\left(T,E_{a}\right)}{1+\tau_{c}{\left(T,E_{a}\right)}^{2}\omega^{2}}$$

where we introduce the anisotropy energy barrier of a SMM *E*_a_. In the presented case of the cobalt-based SMM, the relaxation rate is governed by Raman and Orbach processes as follows:^17^

3 $${\tau_{c}}^{-1}=\underset{\mathrm{Raman}}{CT^{n}}+\underset{\mathrm{Orbach}}{{\tau_{0}}^{-1}\;\exp\left(-E_{a}/k_{B}T\right)}$$

Taking the Raman process into account is essential for an accurate fit of the model, as was shown in a previous study on these SMMs by Rechkemmer et al.^17^ on pressed powder pellets. In general, for different magnetic species, the Orbach process is sufficient for modeling the system.^18,25^ However, in our case, the behavior at LT deviates significantly from an Orbach process, suggesting that a Raman process plays a significant role at LT. According to ref 17, the parameters that fit eq 3 are *C* = 0.088 ± 0.009, *n* = 3.65 ± 0.04, and τ_0_^–1^ = (9.1 ± 0.6) × 10^9^ s^–1^, where the last is known as the inverse attempt frequency. The energy barrier, which was fixed and verified by independent infrared spectroscopy, is *E*_a_ = 230 cm^–1^ = 28.52 meV.^17^ The blocking temperature of the SMM in its bulk form is not reported. However, Rechkemmer et al.^17^ observed magnetic hysteresis at 1.8 K, indicating that the blocking temperature is likely close to this value.

The effect of the SMMs’ noisy spin bath on the relaxation rate of the NV center is incorporated through the coherence signal *e* ^–χ(*t*)^ where χ­(*t*) is known as the coherence function.^32^ This function is dependent on the filter function *F*(ω), which, in turn, is determined by the pulse sequence, and the NSD of the surrounding spin bath *S*(ω) as (derivation in Section S5)^33,34^

4 χ(t)=∫dωS(ω)F(ω)

such that we expect to have a faster relaxation rate as the overlap between the filter function *F*(ω) and the NSD *S*(ω) becomes larger.

In order to explain the influence of the SMMs on *T*_*i*_ (*i* = 1, 2) of the NV center, we use the following equation for the relaxation time dependence on the NSD:^18^

5 $$\frac{1}{T_{i}}={\left(\frac{1}{T_{i}}\right)}_{\mathrm{int}}+{\gamma_{\mathrm{NV}}}^{2}\left\langle B^{2}\right\rangle \int S\left(\omega ,T,E_{a}\right)\;F_{i}\left(\omega \right)\;d\omega$$

where γ_NV_ is the NV electron spin gyromagnetic ratio, $\sqrt{\left\langle B^{2}\right\rangle }$ is the effective magnetic field at the NV position generated by the SMMs, and *F*_*i*_(ω) is the filter function determined by the pulse sequence. In our case, the *T*_1_- and *T*_2_-based protocols are used for characterization of the NSD behavior at the gigahertz and megahertz regimes, respectively.

In a *T*_2_ measurement, the filter function is^25^

$$F_{2}\left(\omega \right)=\frac{32}{\tau }\frac{\sin^{4}\left(\frac{\omega \tau }{4}\right)}{\omega^{2}}$$

where τ/2 is the interpulse delay, as shown in Figure 4 (green curve). According to eqs 2 and 3 and using the fit parameters reported in ref 17, the SMMs’ NSD exhibits a cutoff frequency above the gigahertz range at RT (Figure 4, red curve). At 5 K, this cutoff shifts to lower frequencies, accompanied by an increase in the noise amplitude (purple curve).

**Figure 4:**
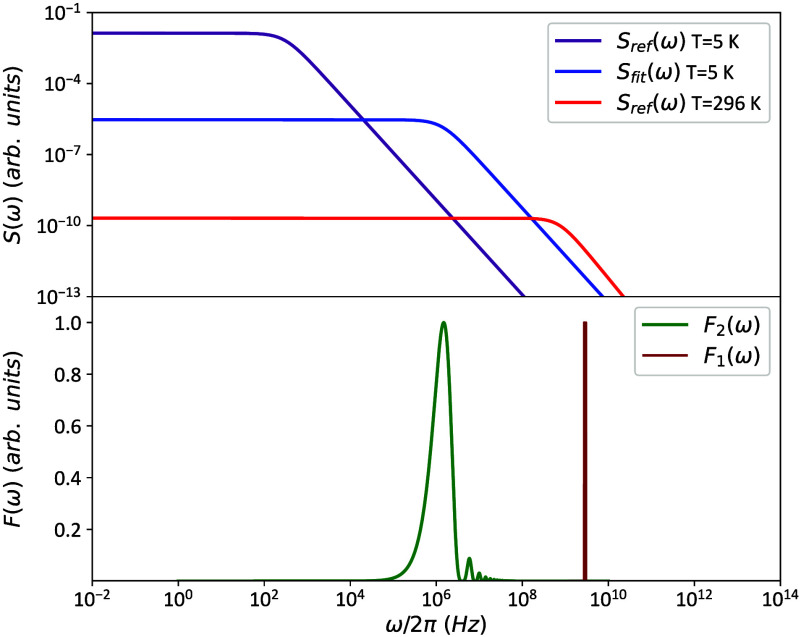
NSDs and filter functions. The upper part depicts the normalized NSD of the cobalt-based SMM at 5 K (purple) and 296 K (red) based on the reference data.^17^ In blue is the fitted NSD at 5 K (for NV22, based on eqs S3 and S4 in Section S6). The bottom part depicts the *T*_2_ (green) and *T*_1_ (brown) filter functions.

We extract the correlation time of the Raman process by fitting the *T*_1_ and *T*_2_ experimental results at 5 K of five NVs in the presence of SMMs. We obtain an average correlation time of τ_c_ = 5 ± 1 μs for these five NVs (see Section S6 for further details). This value is different than a previously published value of τ_c_ = 21 ± 2 ms,^17^ such that the Raman coefficient *C* and power *n* are different in this case.

This result is reasonable due to the different phonon spectra in our measurements. The cobalt-based SMMs, in our case, are not in the bulk state but diluted on a diamond surface (Section S2). The amorphous nature of the drop-cast sample probably leads to distortions, destroying the highly axial nature of the anisotropy, leading to fast underbarrier processes.

Thus, because the Raman process typically involves molecular vibrations in such systems, we obtained a different relaxation rate than that for a bulk sample.^17^ Moreover, varying values of the Raman power *n*, which correspond to differences in relaxation rates, have been previously reported in the literature.^35–37^

In our case, we have a larger overlap at 5 K between the filter function and the NSD in the frequency domain, as can be seen in Figure 4 (blue curve). This accounts for the significant, nearly 1 order of magnitude reduction in *T*_2_ observed in the presence of SMMs. The reduction in *T*_2_ is in contrast to the opposite effect previously observed in bare diamonds, namely, an increase or no change in *T*_2_ when lowering the temperature^38^ because thermal fluctuations of the spin bath decrease with temperature.

In a *T*_1_ measurement, the filter function is given as^25^

$$F_{1}\left(\omega \right)=\underset{i=\pm 1}{\sum }\frac{4\pi /T_{2}^{*}}{{\left(2\pi /T_{2}^{*}\right)}^{2}+{\left(\omega -\omega_{i}\right)}^{2}}$$

where *T*_2_* is the dephasing time of the NV center (∼2 μs) and ω_*i*_ is the resonance frequency of the NV (∼2π × 2.87 GHz at a low magnetic field), as shown in Figure 4 (two sharp, closely spaced brown peaks that appear as a single peak). As a consequence, the spin probe can be sensitive to magnetic field fluctuations in the gigahertz regime.

On the one hand, the dominant *T*_1_ relaxation mechanism of NV centers at RT involves two-phonon Orbach and Raman processes.^39^ On the other hand, at 5 K, the relaxation is temperature-independent and governed by cross-relaxation with neighboring spins. Hence, with our system on bare NVs, we expect to have longer *T*_1_ values at RT than at 5 K, as is also shown in the control measurements of Section S4. However, we observed that in the presence of SMMs, when cooling down from RT to 5 K, we do not obtain a significant difference in the *T*_1_ values, and they remain similar to the RT values of a few hundred microseconds (Figure S4b). Thus, this implies that the SMMs have a major influence on *T*_1_ at 5 K such that relaxation processes induced by the SMMs are significant. This suggests an overlap between the *T*_1_ filter function and the SMMs’ NSD components at both 5 K and RT, as can be seen in Figure 4.

## Magnetic Field Dependence

We performed magnetic-field- and temperature-dependent measurements with SMMs to investigate their effect on the NV center, providing further insight into the SMMs behavior. We observed a field-dependent effect of SMMs on the NV center. As shown in Figure 5a, we measured the *T*_2_ values at different magnetic fields ranging from 18 G up to 62 G in a temperature window ranging from ∼8 K to the base temperature of the experimental setup (see Section S7).

**Figure 5:**
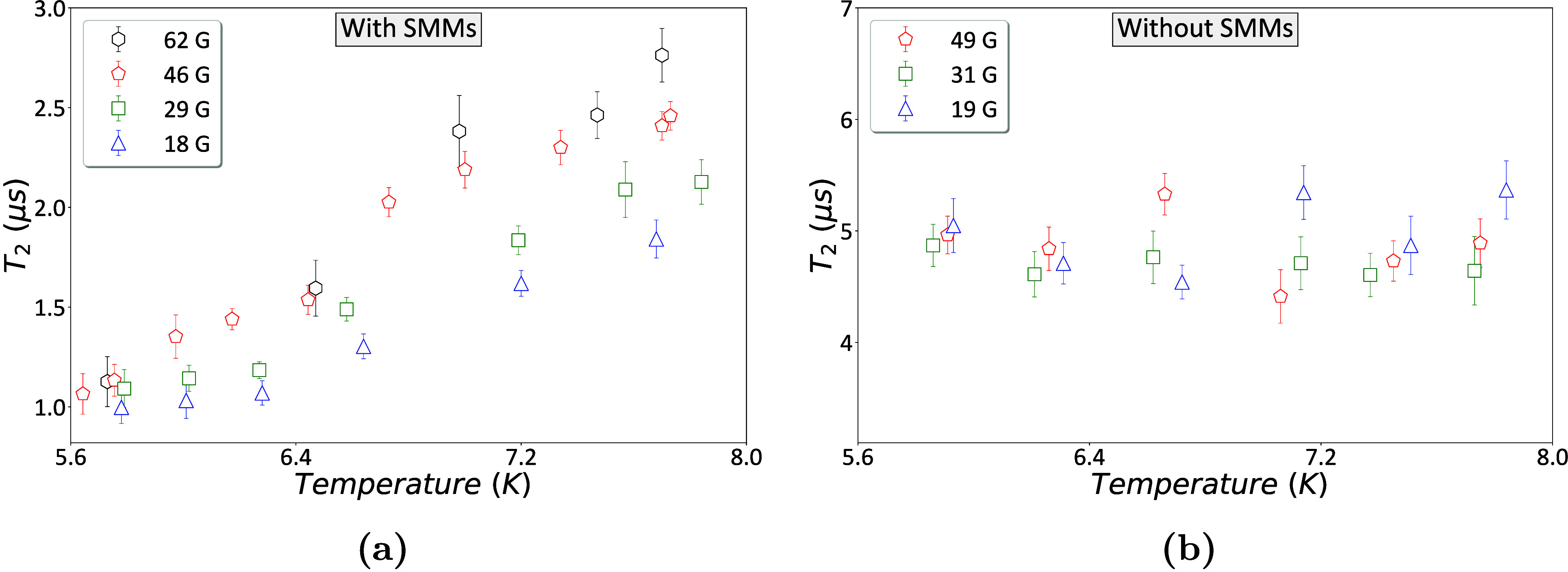
NV coherence *T*_2_ vs temperature at different magnetic fields. Observing the effect of applied magnetic fields on the spin dynamics of cobalt-based SMMs. (a) Data with SMMs. *T*_2_ values of NV22 as a function of the temperature and magnetic field in the presence of SMMs. (b) Reference data without SMMs. *T*_2_ values of NV22 as a function of the temperature and magnetic field in the absence of SMMs. All data were fitted based on Section S3.

Comparing the results, we can observe a significant increase in the coherence time *T*_2_ as the magnetic field increases and also as the temperature increases.

At around 5.6 K, there is almost no change in the coherence times at the different magnetic fields, but as the temperature increases, this change becomes observable. Comparing the *T*_2_ values at about 7.7 K at 18 G (blue) to give *T*_2_ = 1.8 ± 0.1 μs at 62 G (black) to give *T*_2_ = 2.8 ± 0.1 μs, we have an increase of about 60%.

The monotonic increase in *T*_2_ with a rising temperature might be attributed to the reduction in the time-averaged magnetic moments of noise-inducing entities as the temperature moves further above the blocking temperature of the SMMs. Notably, strong temperature dependence in both frequency and magnitude dispersion has been observed in these cobalt-based SMMs during frequency- and temperature-dependent alternating-current magnetic susceptibility measurements.^17^

Reference measurements without SMMs (Figure 5b) suggest that this effect can be attributed to the SMMs. First, the *T*_2_ values are notably longer, consistent with our previous observations. Second, the *T*_2_ values are similar over these temperature and magnetic field ranges.

Thus, this strengthens the fact that the above-mentioned behavior stems from the presence of SMMs.

In conclusion, we presented the sensing of cobalt-based SMMs by using single NV centers. We characterize the magnetic noise spectrum of these molecules at RT and 5 K and under different DC magnetic fields. We observed a significant reduction in the coherence time *T*_2_ and longitudinal relaxation time *T*_1_ of the NV center in the presence of SMMs. We modeled the magnetic noise spectrum of the SMMs as a Gauss–Markov process and used the coherence function to relate the NSD and the filter function to the relaxation profile of the NV center. With this, we could explain the effect of the SMMs spin bath on the *T*_1_ and *T*_2_ relaxation times of the NV centers at different temperatures. Moreover, we were able to extract the correlation time of the SMMs bath at 5 K. By acquiring an additional *T*_1_ and *T*_2_ data set at another LT, it is further possible to determine the Raman coefficient and exponent. In this case, it may be better to work in the diluted regime, where dipolar interactions between the SMMs are less pronounced, because, otherwise, they add another contribution to the extracted correlation time. Our approach represents a novel methodology for extracting the parameters governing the Raman relaxation process of surface-deposited SMMs, which has not been reported in previous studies.

We have also observed a significant variation in the *T*_2_ of an NV center as a function of temperature in the presence of SMMs, particularly near the blocking temperature regime. In addition, a strong positive influence of an applied magnetic field on *T*_2_ was observed in this temperature regime. This indicates that applying a direct-current magnetic field modulates the noise profile of the SMMs, a noteworthy observation with potential implications for their use in storage technology. Going to even lower temperatures, i.e., below the blocking temperature, would allow one not only to identify the structural form of the molecules next to the NV on the surface of the diamond, i.e., whether it is a molecular crystal or isolated molecules, but also to isolate the contribution of SMMs’ intermolecular dipolar coupling, which is currently difficult to establish.

The method we presented here can help in the research and development of SMMs because we can sense them at the surface, at different temperatures, and at nanoscopic volumes. Moreover, this method can also be applied to the detection and characterization of other types of SMMs, as well as potential molecular qubits.

## Supplementary Material



## Data Availability

The data that support the findings of this study are openly available at the following DOI: 10.34933/400b149f-da31-441b-aace-0e4b037205b8.

## Notes

Simulation of NV effective sensing radius and volume (Section S1), comparison between bulk and drop cast SMMs (Section S2), *T*_1_ and *T*_2_ curves fitting (Section S3), additional statistics and control measurements (Section S4), derivation of the coherence function in the frequency domain (Section S5), model fitting to the data (Section S6), temperature control at LT (Section S7), sample preparation (Section S8), SMMs overview (Section S9), XPS analysis (Section S10), experimental setup (Section S11), ab initio calculations (Section S12), T_1_ protocol (Section S13), and quantum sensing with NV centers (Section S14) (PDF)

## ‡

School of Physics and Astronomy, Shanghai Jiao Tong University, Shanghai 200240, China
