# Supplementary material for: Sensing Single-Molecule Magnets with Nitrogen-Vacancy Centers
Source: Nano Lett. 2026 Jan 29;26(5):1655–61. doi: 10.1021/acs.nanolett.5c05066 (PMC12904096; doi:10.1021/acs.nanolett.5c05066)
Supplement: Supplementary file 1 [file nl5c05066_si_001.pdf]

Supplementary Information

Sensing single-molecule magnets with nitrogen vacancy centers

Ariel Smooha[1,†], Jitender Kumar[1,†], Dan Yudilevich[1], John W. Rosenberg[1], Valentin Bayer[2], Rainer Stöhr[3], Andrej Denisenko[3], Tatyana Bendikov[4], Anna Kossoy[4], Iddo Pinkas[4], Hengxin Tan[5,‡], Binghai Yan[5], Biprajit Sarkar[6,7], Joris van Slageren[2], and Amit Finkler[1,*]

[1]Department of Chemical and Biological Physics, Weizmann Institute of Science, 7610001 Rehovot, Israel
[2]Institute of Physical Chemistry, University of Stuttgart, Pfaffenwaldring 55, 70569 Stuttgart, Germany
[3]Third Institute of Physics, IQST and ZAQuant, University of Stuttgart, 70569 Stuttgart, Germany
[4]Department of Chemical Research Support, Weizmann Institute of Science, Rehovot 7610001, Israel
[5]Department of Condensed Matter Physics, Weizmann Institute of Science, Rehovot 7610001, Israel
[6]Institute of Inorganic Chemistry, University of Stuttgart, Pfaffenwaldring 55, 70569 Stuttgart, Germany
[7]Institut für Chemie und Biochemie, Freie Universität Berlin, 14195 Berlin, Germany

[†]These authors contributed equally to this work

[‡]Present address: School of Physics and Astronomy, Shanghai Jiao Tong University, Shanghai, China
[*]amit.finkler@weizmann.ac.il

Contents

S1 Simulation of NV effective sensing radius and volume 3
S2 Comparison between bulk and drop cast SMMs 5
S3 T_1 and T_2 curves fitting 8
S4 Additional statistics and control measurements 9
S5 Derivation of the coherence function in the frequency domain 12
S6 Model fitting to the data 13
S7 Temperature control at LT 15
S8 Sample preparation 16
S9 SMMs overview 18
S10 XPS analysis 19
S11 Experimental setup 20
S12 ab-initio calculations 21
S13 The T_1 protocol 24
S14 Quantum sensing with NV centers 25

S1 Simulation of NV effective sensing radius and volume

To estimate the NV sensing volume for surface-deposited spins, we used a spherical-cap geometry corresponding to an NV depth of 8 nm below the diamond surface. The NV center was assumed to semi-classically detect magnetic fields from nearby spins with positions randomly distributed within a sphere of radius 35 nm. For a given realization, the magnetic moments of the spins were assigned random orientations, and the total magnetic field projected along the NV axis was calculated by summing the dipolar contributions from all spins within a series of radii. This procedure was repeated over 200 realizations to account for fluctuations of the spins. From the radial dependence of the projected field, an effective sensing radius of ∼20 nm was determined, corresponding to the region that contributes significantly to the NV signal (see Fig. S1). The sensing volume, which has the geometry of a spherical cap, can be calculated as

$$V_{sensing} = \frac{\pi(R-d)^2(2R+d)}{3}$$

where d ($\sim$ 8 nm) is the NV depth and R is the sphere radius. Thus, for a sensing radius of 20 nm, which captures most of the signal contribution, the corresponding sensing volume is on the order of $\sim$ (20 nm)^3. Comparable NV sensing ranges were likewise observed for transition-metal sensing [1].

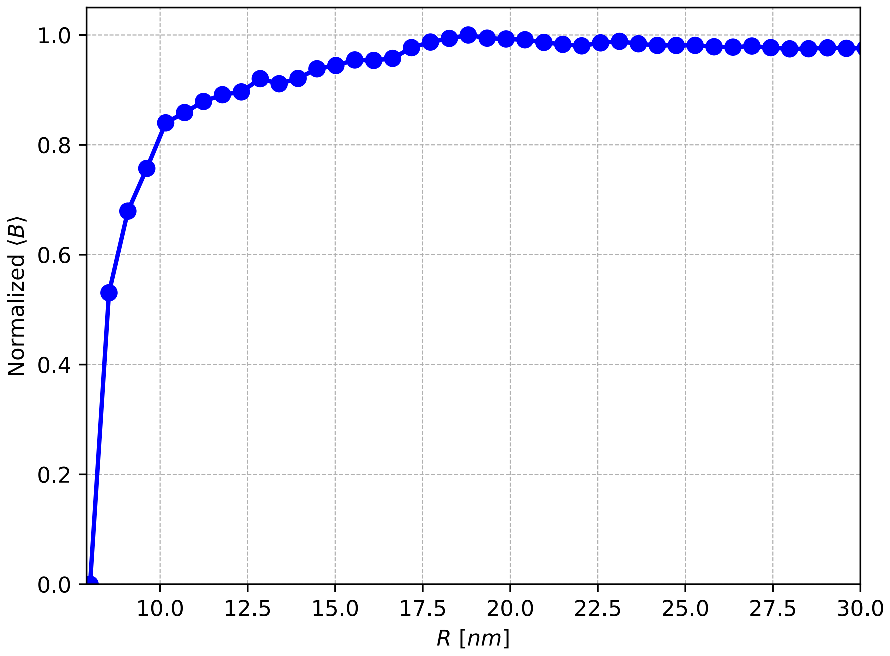

Figure S1: Effective sensing radius simulation. Normalized magnetic field amplitude as a function of the sensing radius of the NV center.

In the liquid state with a concentration of 1 mM, at least 4 molecules are to be sensed. In practice, however, the solution evaporates, resulting in a significantly higher concentration of single-molecule magnets (SMMs) on the surface. We drop-cast about 2 µl of the SMM solution. Thus, overall, we have about $1.2 \cdot 10^{15}$ SMMs in this volume. Assuming the formation of a uniform layer over the diamond (2 mm × 2 mm), and a molecule diameter of about 2 nm, we expect to have $\pi \left(\frac{2\ \text{nm}}{2\ \text{mm}}\right)^2 \times 1.2 \cdot 10^{15} = 3.8 \times 10^3$ molecules per cross-section, corresponding to a layer thickness of about ~ 7.6 µm. Thus, even if a few factors are missing, the calculation still indicates that the sensing volume is completely saturated with molecules. It is also expected that the layer is not uniform, but the variations would fluctuate around this value and would not approach the scale of the sensing volume. Therefore, assuming a saturation of the sensing volume, we obtain

$$n_{\text{sense}} \sim \frac{3}{4\pi} \left(\frac{20\ \text{nm}}{2\ \text{nm}}\right)^3 = 239$$

such that approximately 239 SMMs contribute to the measured signal within the corresponding sensing volume.

We should note that at such intermolecular spacing of ∼2 nm, dipolar interactions between neighboring SMMs are significant (∼tens of MHz) and can induce spin flip-flop processes [2]. In addition, at very short intermolecular distances, weak exchange interactions cannot be strictly excluded and may further contribute to the NSD. However, since the cobalt ion cores are separated by organic ligands, such interactions are expected to be suppressed, making direct exchange less probable and significant than dipolar coupling. Importantly, while dipolar interactions modify the timescale of the magnetic fluctuations, they are intrinsically temperature independent and therefore do not account for the observed thermally activated behavior of the noise spectrum as we cool the system from RT to 5 K, as discussed in the main text.

S2 Comparison between bulk and drop cast SMMs

To verify that the SMMs remain unchanged after drop-casting, we also employed Raman spectroscopy, which utilizes a confocal microscope (LabRAM HR, HORIBA, 532nm laser) that enables diffraction-limited spatial resolution. Using the Raman system, we measured the Raman spectrum of drop-cast SMMs (from a 1 mM solution in acetonitrile) on glass and diamond, comparing it with the bulk sample. From Fig. S2 one can observe the very strong similarity between the signals. For the thin layer of the SMM on diamond (brown line), one can observe the dominant peaks at wavenumbers of approximately 1400 cm^{-1} and 3150 cm^{-1}, which originate from the CVD-grown diamond. In any case, all other peaks closely resemble those of the bulk crystal (purple line). Raman spectra of a thick SMMs layer on diamond (green line) and drop-cast SMMs on glass (blue line) were also measured, with the latter serving to isolate the SMMs signal from the strong diamond Raman background. Additionally, the spectra here closely resemble those of the bulk crystal. These observations strongly indicate that the drop-cast SMMs preserve their chemical composition in all cases, while their intermolecular framework becomes more amorphous and disordered, thereby affecting Raman phonon features.

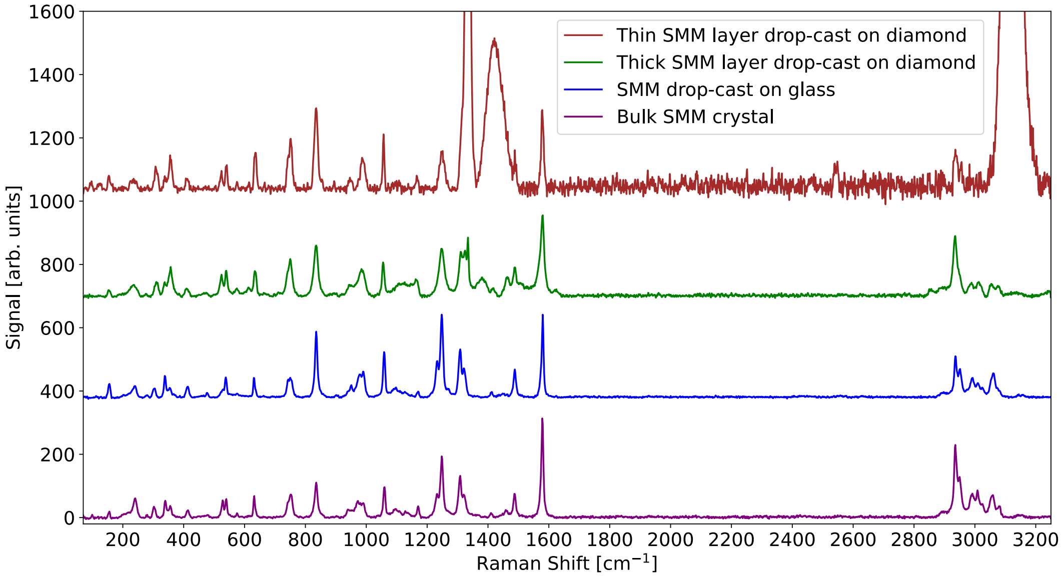

Figure S2: Raman measurements for the bulk and drop-cast SMMs. Raman spectrum for the bulk crystal (purple), drop cast SMM on glass (blue), thick drop cast SMM layer on diamond (green), and thin SMM layer on diamond (brown). For the latter, we obtain a strong truncated signal from the diamond surface. The data has been baseline corrected by using a rolling ball algorithm.

We have also performed GIXRD (grazing incidence X-ray diffraction) analysis on drop-cast SMMs as well as standard measurements of the bulk powder sample. We show the

simulated powder pattern based on the single crystal structure provided by Ref. 3 in Fig. S3a (magneta) in comparison to the powder used in this work (black), which was synthesized for us in Stuttgart. The left shift (toward larger d-spacings) observed in the experimental powder pattern is due to thermal expansion since single crystal data was collected at 100 K (see Ref. 3, SI Table 1) while the powder was measured at RT. Excess of intensity in some peaks is due to coarse crystal size of the powder. We have also performed GIXRD analysis of the drop-cast material on a diamond substrate, shown in Fig. S3b (black pattern). For reference, the powder diffraction pattern of the bulk SMM powder is also shown (red pattern). The measurements reveal that the film consists of crystals with sizes ranging from 60 to 130 nm, with their peaks closely matching those of the powder, confirming the SMM structure. A wide hump at $2\theta \approx 9°$ appears at the position of the main peak belonging to the crystalline phase, which implies that there is also a nano-crystalline/disordered fraction of the same material. Indeed, the peak at $2\theta \approx 9°$ is higher than expected for a random powder pattern. This can be explained by anisotropic crystal morphology and crystal orientation within the film.

Measurements were performed with a TTRAX Rigaku Japan diffractometer equipped with a rotation anode at 10 kW. For GIXRD the parallel beam was shaped by a CBO (cross-beam optics) and a long 0.114° degree PSA (parallel slit analyzer). A graphite monochromator was installed before the scintillator detector. Powder was measured with focused beam in Bragg-Brentano geometry with 5° degree Soller slits installed before and after the sample and graphite monochromator installed before detector.

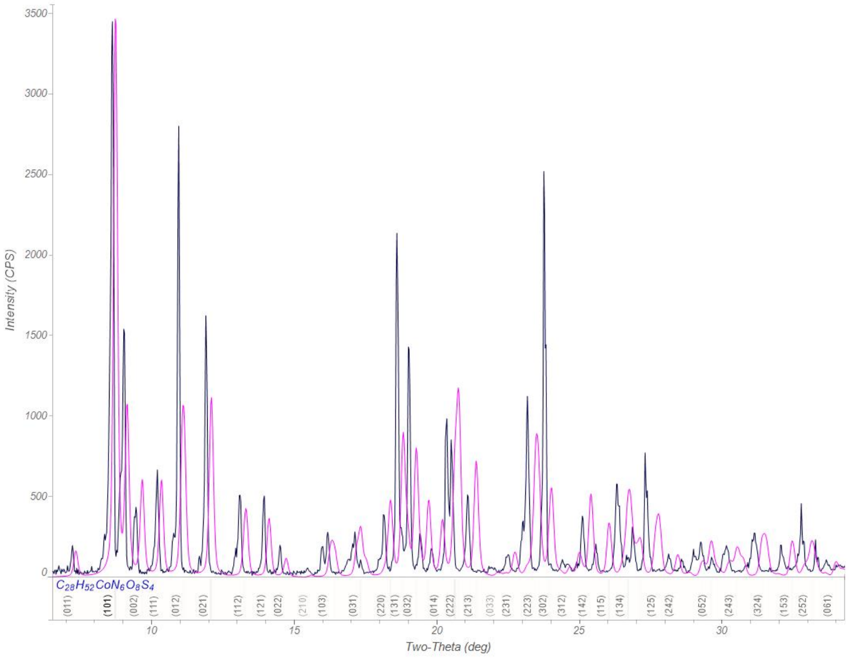

(a)

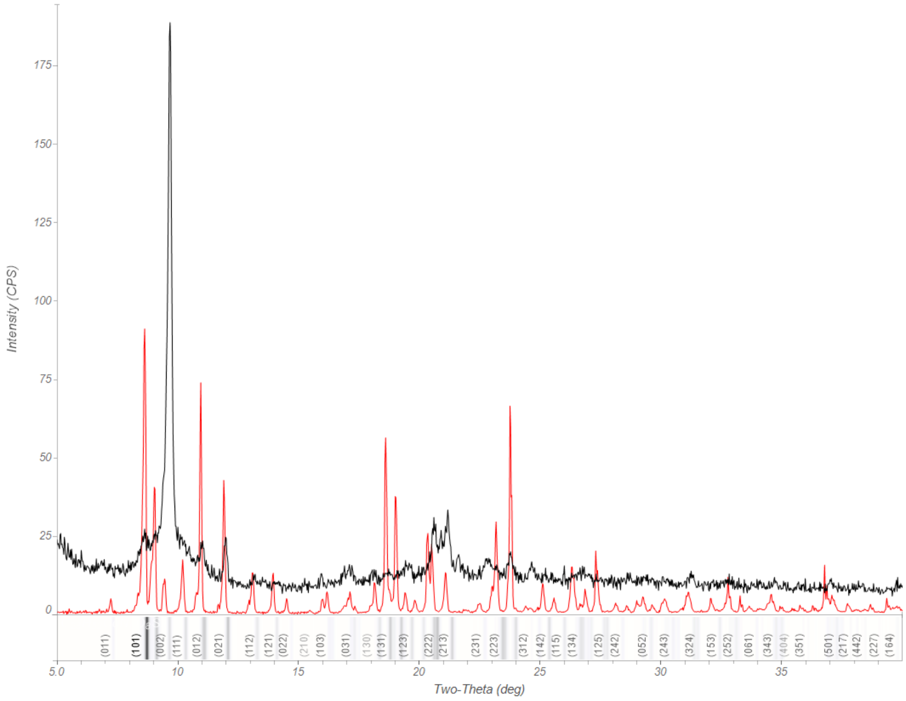

(b)

Figure S3: XRD measurements for the bulk and drop-cast SMMs. (a) Simulated pattern for SMM based on single crystal structure file [3] (magneta, 100 K) and experimental pattern of the powder used in this work (black, RT). (b) XRD for the bulk crystal powder (red) and GIXRD of drop-cast SMMs on diamond measured at an incident angle of $\alpha = 1°$ (black, vertically offset for clarity). Both measured at RT.

S3 T_1 and T_2 curves fitting

During a measurement with a single NV center, we collect the emission during the first 200 ns out of a 2 µs laser pulse. In the averaged readout of many iterations, the number of photons collected by the APDs is counted. Thus, for each evolution time τ in a T_1 or T_2 pulse sequence, we count the total number of photons. To fit the T_2 data, we measured the relaxation profiles of the superposition state using $(\pi/2)_x$ and $(\pi/2)_{-x}$ operations to map the superposition state to longitudinal states. We then subtracted the two curves and fitted the resulting data to a stretched exponential function,

$$S_2(t) = A_2 \exp\left(-\frac{t}{T_2}\right)^n \tag{S1}$$

from which we extract the relaxation time T_2. Then, to normalize the data (shown in Fig. 3a of the main text, we divided it by the maximal value.

For a T_1 measurement, we tracked the relaxation profile from the $|0\rangle$ state. In this case, we fitted the data to a decaying exponential

$$S_1(t) = A_1 \exp\left(-\frac{t}{T_1}\right) + C_1 \tag{S2}$$

In order to normalize the data (shown in Fig. 3b of the main text, we used the fitted offset parameter C_1 to obtain the fractional contrast

$$S_{\text{contrast}}(t) = \frac{S_1(t) - C_1}{A_1}$$

Then, we fitted the obtained graph $S_{\text{contrast}}(t)$ to a decaying exponential, according to Eq. S2, and divided the fit by the extracted amplitude to obtain the normalized signal $S_{\text{normalized}}(t)$. The data profile and the T_1 remained unchanged, confirming the reliability of the normalization procedure [4].

S4 Additional statistics and control measurements

In order to validate the T_1 and T_2 results in the main text and have a broad set of measurements, we performed the experiments on a set of NVs. We performed measurements at 296 K (RT) and 5 K (LT) and also with and without the cobalt-based SMMs. In the presence of SMMs, we detected a significant decrease in the T_2 coherence times when moving from RT to LT, as shown in Fig. S4a. We observed a modest increase in T_1 values, much less than expected over this temperature difference for bare NVs (Fig. S4b). These results are discussed in the main text.

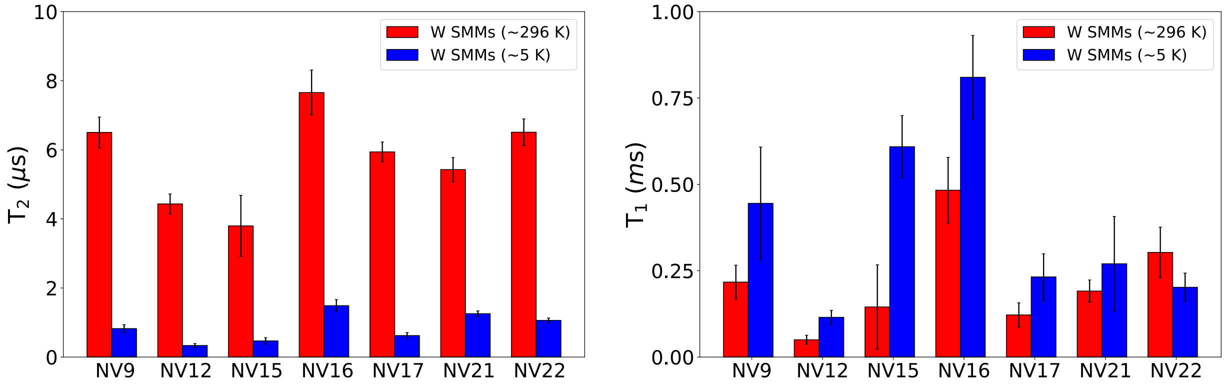

(a) **(b)**

Figure S4: The effect of the cobalt-based SMMs at RT and LT. **(a)** T_2 values for different NVs at 296 K (red) and 5 K (blue). **(b)** T_1 values for different NVs at 296 K (red) and 5 K (blue).

In order to verify that the effect stems from the interaction of the NV center with the cobalt-based SMMs spin bath, we also measured the relaxation profiles with and without SMMs at 5 K. As can be seen in Fig. S5a, there is a significant decrease in the T_2 coherence times in the presence of SMMs. Moreover, comparing the T_1 relaxation times, there is a decrease of about an order of magnitude in the presence of SMMs (Fig. S5b).

As control measurements, we present results for T_2 and T_1 values with and without SMMs at RT for the same diamond (Figs. S6a and S6b) and for a different diamond membrane cut from the same single crystal and prepared using the same processes (Figs. S7a and S7b respectively). On the one hand, one can observe the decrease of the T_1 relaxation times in the presence of SMMs. On the other hand, there seems to be no effect of the SMMs on T_2 coherence times, in stark contrast with the low temperature data (Fig. S5). Another control measurement comparing the diamond without the SMMs at different temperatures is shown in Fig. S8, showing, as expected for bare NVs, an increase of T_1 as the temperature decreases [5] and essentially little or no change in T_2. The latter is in comparison to the unambiguous, much significant effect of the SMMs on the NVs T_2 values, shown in Fig. S4a.

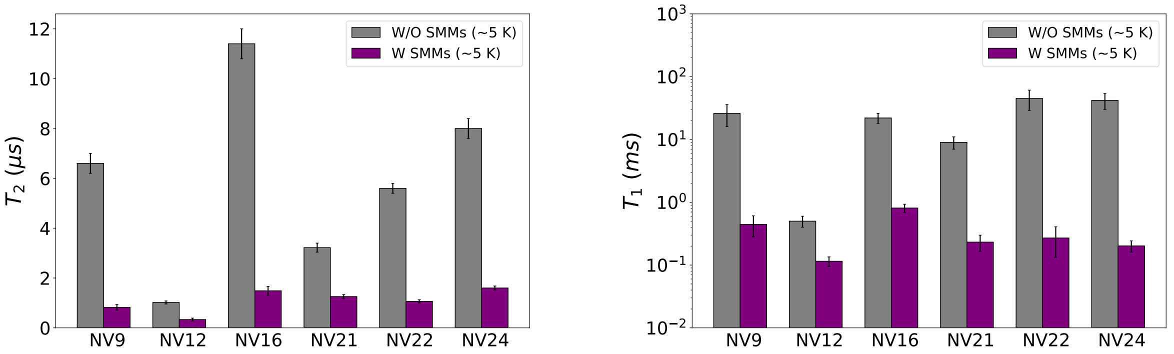

(a) **(b)**

Figure S5: The effect of the cobalt-based SMMs at low temperature (5 K). **(a)** T_2 values for different NVs at 5 K in the presence (purple) and absence (gray) of SMMs. **(b)** T_1 values for different NVs at 5 K in the presence (purple) and absence (gray) of SMMs.

We note that NV12 is anomalous (also in model fitting, Sec. S6); however, for completeness, we disclose all relevant information.

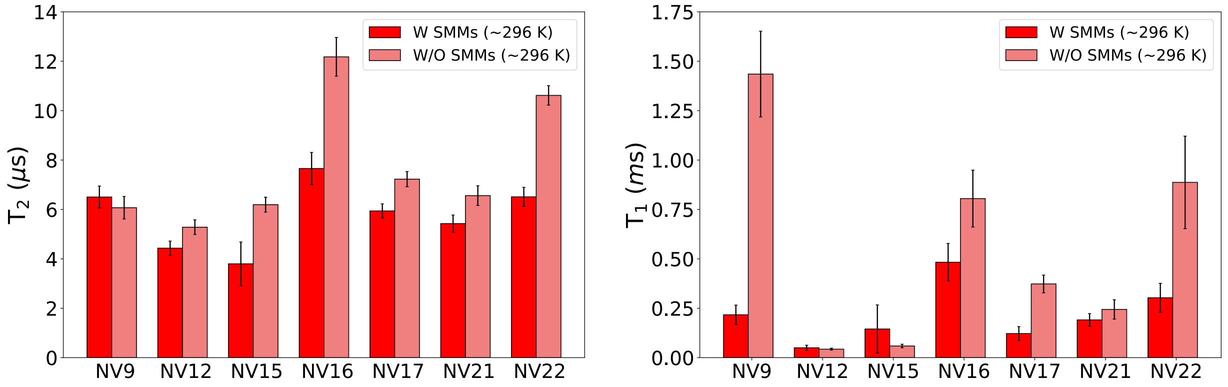

(a) **(b)**

Figure S6: The effect of the cobalt-based SMMs on the relaxation times at RT. **(a)** T_2 values for different NVs with (red) and without (light coral) SMMs. **(b)** T_1 values for different NVs with (red) and without (light coral) SMMs.

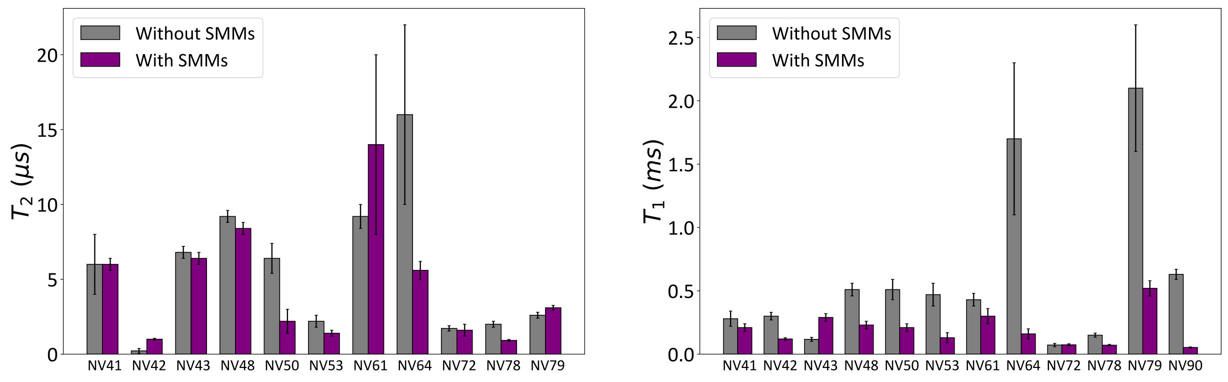

(a) **(b)**

Figure S7: The effect of the cobalt-based SMMs on the relaxation times at RT (a different diamond membrane). **(a)** T_2 values for different NVs with (purple) and without (gray) SMMs. **(b)** T_1 values for different NVs with (purple) and without (gray) SMMs.

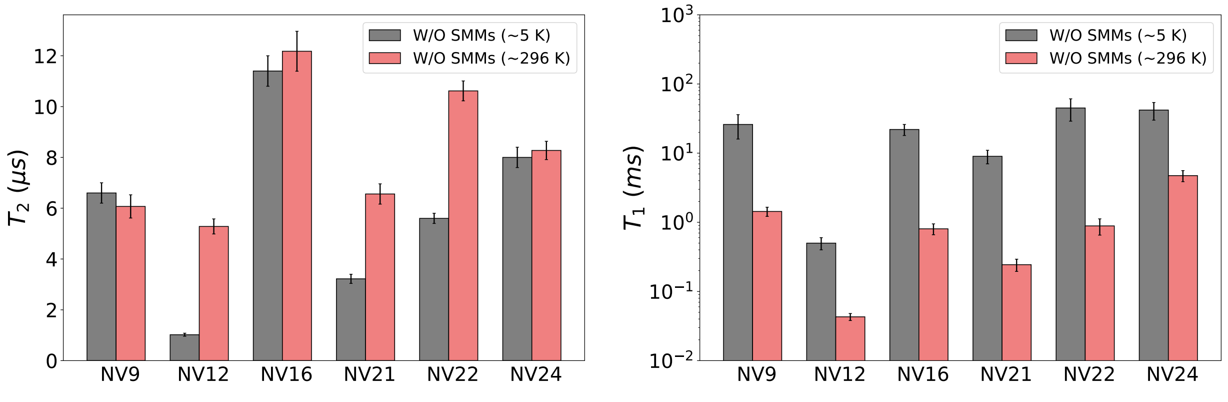

(a) **(b)**

Figure S8: The effect of temperature on the relaxation times (without SMMs). **(a)** T_2 values for different NVs without SMMs at 296 K (light coral) and 5 K (gray). **(b)** T_1 values for different NVs without SMMs at 296 K (light coral) and 5 K (gray).

S5 Derivation of the coherence function in the frequency domain

Here, we use $\eta(t';t) = \int f(\omega)e^{i\omega t'}d\omega$ and $\varepsilon(\tau) = \int S(\omega)^{i\omega t'}d\omega$ to derive Eq. 4 in the main text. It is performed by moving from the NSD and the filter function in the time domain, to their frequency domain. The time domain form is given by Ref. 6 assuming a Gauss-Markov noise source

$$
\begin{aligned}
\chi(t) &= \int_0^t \int_0^t \eta(t';t)\eta(t'';t)\varepsilon(\tau)dt'dt'' \\
&= \frac{1}{(2\pi)^3}\int_0^t \int_0^t \int f(\omega')e^{i\omega' t'}d\omega' \int f(\omega'')e^{i\omega'' t''}d\omega'' \int S(\omega)e^{i\omega(t''-t')}d\omega dt'dt'' \\
&= \frac{1}{(2\pi)^3}\int\int\int\int\int f(\omega')e^{i\omega' t'} f(\omega'')e^{i\omega'' t''} S(\omega)e^{i\omega(t''-t')}d\omega d\omega' d\omega'' dt'dt'' \\
&= \frac{1}{(2\pi)^3}\int\int\int\int\int f(\omega')f(\omega'')S(\omega)e^{it'(\omega'-\omega)}e^{it''(\omega''+\omega)}d\omega d\omega' d\omega'' dt'dt'' \\
&= \frac{1}{2\pi}\int\int\int f(\omega')f(\omega'')S(\omega)\delta(\omega'-\omega)\delta(\omega''+\omega)d\omega d\omega' d\omega'' \\
&= \frac{1}{2\pi}\int\int f(\omega')f(-\omega)S(\omega)\delta(\omega'-\omega)d\omega d\omega' \\
&= \frac{1}{2\pi}\int f(\omega)\underbrace{f(-\omega)}_{=f(\omega)^*}S(\omega)d\omega \\
&= \frac{1}{2\pi}\int d\omega \left|f(\omega)\right|^2 S(\omega) \\
&= \frac{1}{2\pi}\int d\omega F(\omega)S(\omega).
\end{aligned}
$$

From this equation, the role of the pulse sequence becomes clear such that given some noise spectrum density $S(\omega)$, the pulse sequence acts as a filter function $F(t,\omega)$, and changing the pulse sequence modifies the spin probe sensitivity to different frequency components of the noise.

S6 Model fitting to the data

In order to fit our model to the experimental results, we calculated the overlap between the SMM NSD and the T_1 and T_2 filter functions as explained in Eq. 4 and Eq. 5 of the main text. We performed this fitting for the ~5 K data from NV24, NV22, NV21, NV16, NV12 and NV9 in the presence and absence of SMMs (see Table S1). We assume to have an intrinsic magnetic noise from spin species in the bare diamond and an external noise from the SMMs. For an equilibrium Markovian noise source, according to Eq. 4 of the main text and the Redfield theory [7], we get the next equation for T_1

$$\frac{1}{T_1} = \sum_i \frac{\Delta^2 \left(\frac{1}{\tau_c} + \Delta^2 \tau_c\right)}{\left(\frac{1}{\tau_c} + \Delta^2 \tau_c\right)^2 + \omega_i^2} \tag{S3}$$

where $\Delta^2 = \gamma_e^2 \langle B^2 \rangle$ corresponds to the variance of the magnetic field fluctuations, τ_c is the correlation time of the noise bath, and $\omega_{i=\pm 1}$ is the NV resonance frequency. For the T_2 data, we used a derivation based on Ref. 8 by *de Sousa*, where the Hahn echo signal for an equilibrium Markovian noise source is given as

$$\langle S_z(t) \rangle = e^{-\chi(2\tau)} = \exp\left(-\Delta^2 \tau_c^2 \left[\frac{2\tau}{\tau_c} + 4e^{-\tau/\tau_c} - e^{-2\tau/\tau_c} - 3\right]\right) \tag{S4}$$

where $\chi(t)$ is the coherence function and τ is the inter-pulse delay. Using global fitting with Eq. S3 and Eq. S4, we managed to extract the values of the free parameters for the different NVs. We performed simultaneous fitting using the T_1 and T_2 data from the different NVs in the presence and absence of SMMs at ~5 K (see fit results at Table S1), using Eq. 8 and Eq. 9 of the main text. An example of the normalized fitted curves of NV22 is shown in Fig. S9. From the fit, we could calculate the parameters of the Raman process (Eq. 3 of the main text), which is the dominant relaxation process at ~5 K. However, for extracting the power n and the coefficient C of the Raman process, one has to acquire the T_1 and T_2 data in at least two distinct low temperatures, which in our case amounts to the dataset at ~5 K due to hardware limitations. In this case, assuming the Raman coefficient remains the same as the literature one ($C = 0.088 \pm 0.009$), we obtain $n = 8.5$. Nevertheless, the coefficient C might differ. Thus, as far as we are aware, this method introduces a novel approach for extracting the parameters governing the Raman relaxation process, which has not been reported in prior studies.

Table S1 summarizes the extracted parameters for the different NVs that were measured with and without SMMs at ~5 K. In the table, Δ_{diamond} and τ_{diamond} denote the variance and correlation time for the bare diamond, whereas Δ_{SMM} and τ_{SMM} denote the variance and correlation time for the SMMs. The correlation times of the SMMs τ_{SMM} were extracted to be on the same order and similar in all cases, but for NV12, where the SNR for the T_2 data

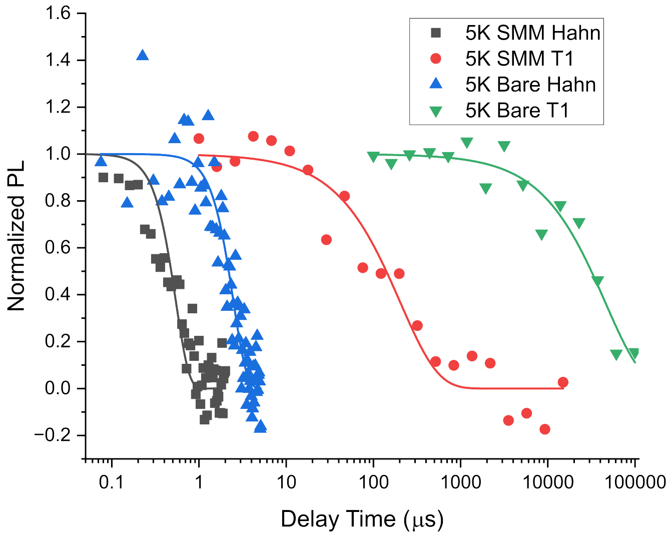

Figure S9: Global fitting for NV22 at ∼5 K. T_2 model fitting with (dark gray) and without (blue) SMMs. T_1 model fitting with (red) and without (green) SMMs. From the global fit we have $\Delta_{\text{diamond}} = 1.31 \pm 0.04$ MHz, $\Delta_{\text{SMM}} = 6.8 \pm 0.3$ MHz, $\tau_{\text{diamond}} = 15 \pm 2$ µs, and $\tau_{\text{SMM}} = 4.6 \pm 0.5$ µs as shown in Table S1.

with SMMs was not good (to have $r^2 = 0.4618$), and thus the overall fit is not good and the error in the SMM's parameters is significant. Therefore, the average correlation time τ_{SMM} of $= 5 \pm 1$ µs given in the main text excludes NV12. Rather than that, based on the fit, we can assume a relatively uniform SMM environment in the NVs regime (an area of hundreds of square microns). The bare diamond parameters are different due to the different bare environments across NVs, corresponding to their T_1 and T_2 values without SMMs.

NV center	τ_{SMM} (µs)	Δ_{SMM} (MHz)	τ_{Diamond} (µs)	Δ_{Diamond} (MHz)	R-squared
NV24	8.2 ± 0.7	5.2 ± 0.2	42 ± 4	1.04 ± 0.04	0.9154
NV22	4.6 ± 0.5	6.8 ± 0.3	15 ± 2	1.31 ± 0.04	0.8638
NV21	5.9 ± 0.7	6.0 ± 0.3	11 ± 2	2.2 ± 0.1	0.8944
NV16	5.4 ± 0.7	4.6 ± 0.2	105 ± 13	0.97 ± 0.05	0.8417
NV12	582 ± 289	416 ± 115	2.8 ± 0.8	6.2 ± 0.6	0.6625
NV9	1.8 ± 0.3	7.1 ± 0.4	33 ± 4	1.24 ± 0.06	0.8390

Table S1: Extracted variance of magnetic fluctuations $\Delta^2 = \gamma_e^2 \langle B^2 \rangle$ and correlation time τ for the different NV centers at ∼5 K based on the acquired data with and without SMMs. Δ_{diamond} and τ_{diamond} denote the variance and correlation time for the bare diamond, whereas Δ_{SMM} and τ_{SMM} denote the variance and correlation time for the SMMs.

S7 Temperature control at LT

In order to control the temperature at LT (ranging from 5 K to 10 K due to MW heating), we modified the duty cycle of a sequence by implementing a wait time inside the sequence. The temperature increases with shorter wait times due to the higher average MW power input. However, during the wait time, the optical spin contrast decreases due to a decay governed by depolarizing and dephasing channels. Thus, using this method to control the temperature during a spin echo sequence had to be verified so as not to affect the end result of a measurement, namely, the measured T_2 value. Therefore, this was verified with a simulation where we introduced a wait time to the spin echo sequence and fit the resultant decay to extract the T_2 value. Transverse and longitudinal relaxation processes were taken into account through jump operators in the Lindblad master equation for open systems, as follows

$$\dot{\rho}(t) = -\frac{i}{\hbar}\left[H(t), \rho(t)\right] + \sum_n \frac{1}{2}\left[2C_n\rho(t)C_n^\dagger - \rho(t)C_n^\dagger C_n - C_n^\dagger C_n \rho(t)\right]$$

with $C_1 = \sqrt{\frac{1}{T_2}}\hat{S}_z$, $C_2 = \sqrt{\frac{1}{T_1}}\hat{S}_x$, and $C_3 = \sqrt{\frac{1}{T_1}}\hat{S}_y$. From the simulation depicted in Fig. S10, one can observe the significant discussed reduction in contrast when using a wait time of 50 µs. However, the result showed only a 5.1% difference in the extracted T_2 value.

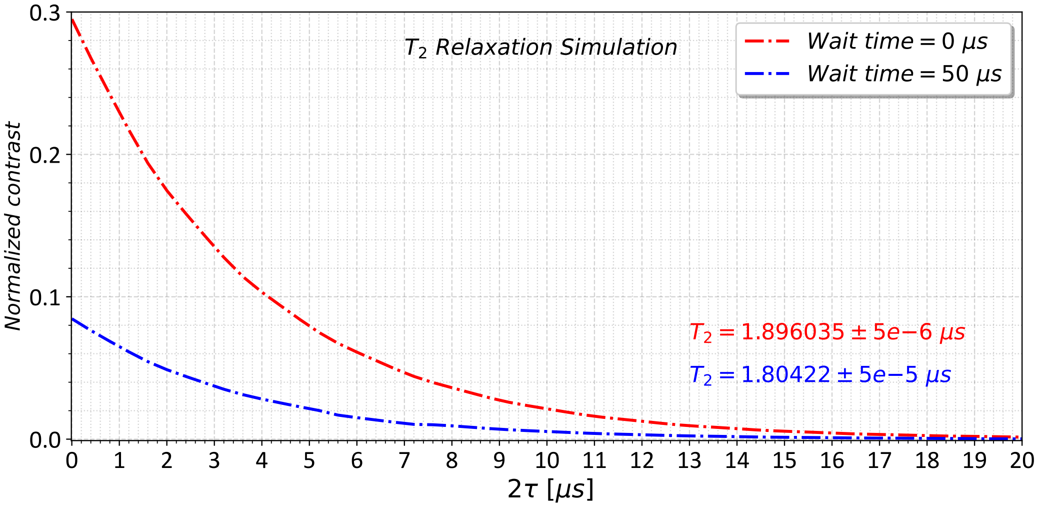

Figure S10: Temperature control at LT (5 to 10 K). A simulation of T_2 curves at different wait times implemented in the spin echo pulse sequence for temperature control by duty cycle determination. The two curves are for no wait time (red) and for 50 µs wait time (blue).

S8 Sample preparation

The SMM, $(HNEt_3)_2[Co^{II}(L^{2-})_2]$, where the ligand of the inorganic complex L stands for 1,2-bis(methanesulfonamido)benzene was synthesized as described in Ref. 3. The diamond sample used in this work is a thin, single-crystal [100] diamond membrane (approximately 30 µm thick) patterned with nanopillar diamond waveguides. Shallow NV centers were created in the diamond by $^{15}N^{+}$ nitrogen ion implantation with and energy of 5 keV. Afterwards, the sample was annealed in high vacuum at a temperature of 950°C for two hours. Due to channeling effects during ion implementation, the NV depths can vary with an implantation depth of 8 ± 3 nm. This is based on Stopping and Range of Ions in Matter (SRIM) [9] simulation shown in Fig. S11a. In the sample, there is an average of 1 NV per 1 μm^2. We performed the measurements using several single NV centers in nanopillars ($\sim$ 500 nm diameter). To verify that each NV center is a single emitter, we recorded the second-order photon autocorrelation function $g^{(2)}(\tau)$. A value of $g^{(2)}(\tau) < 0.5$ for $\tau \to 0$ is an indication for the detection of a single photon emitter [10].

After depositing the SMMs on the diamond membrane using a few drops of a 1 mM solution (in acetonitrile) of the cobalt based SMM (Sec. S9) we could observe the pinkish color of the membrane under the optical microscope (Fig. S11b). We used this concentration of 1 mM as it was used in magnetic circular dichroism measurements in Ref. 3. We also verified the presence of the SMMs with X-ray photoelectron spectroscopy (XPS), from which we can gain information regarding elemental content, functional group and oxidative state (Sec. S10). Note that we first measured the NV centers with the drop-cast SMMs and performed the control measurements only after acid cleaning. Starting with bare NVs would not guarantee that SMMs remain after drop-casting, making the workflow unreliable. Although cleaning the sample afterward is not ideal, this procedure ensured consistent SMM coverage for the initial measurements.

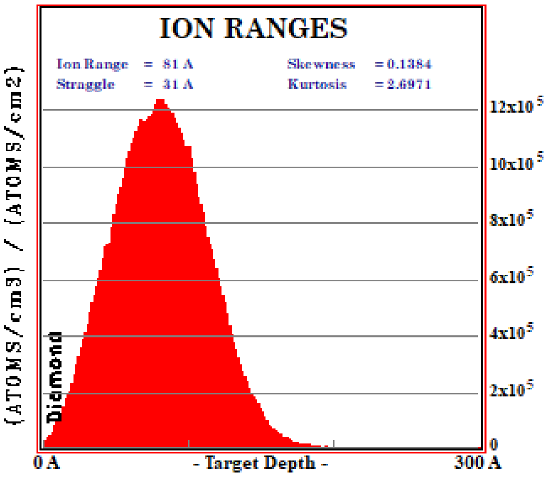

(a)

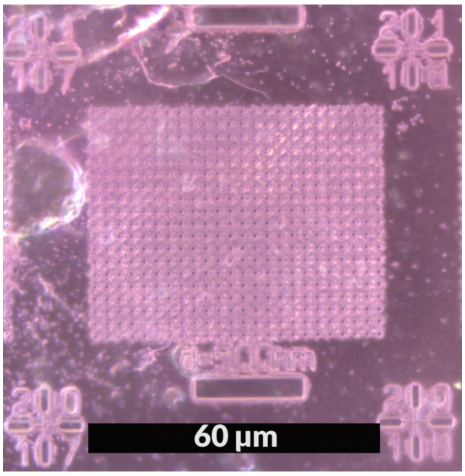

(b)

Figure S11: Diamond sample. **(a)** SRIM simulation for depth profile of implanted NVs at 5 keV energy. **(b)** A micrograph ($\sim$ 80 µm width) of the diamond membrane, with an array of nanopillars, after drop casting cobalt-based SMMs. It has a pinkish color similar to that of the bulk material.

S9 SMMs overview

The SMM is a cobalt based molecule (Fig. S12a) which is defined as a Kramers system with $S = 3/2$ manifold (Fig. S12b). The effective energy barrier in this case is $U_{\text{eff}} = 2|D| = 230\,\text{cm}^{-1} = 28.5\,\text{meV}$. We can not address the $|m_s = \pm 1/2\rangle \rightarrow |m_s = \pm 3/2\rangle$ transition, but only the $|m_s = -1/2\rangle \rightarrow |m_s = +1/2\rangle$ transition. The former transition is in the infrared (IR) regime and can be observed with IR spectroscopy, while the latter is not observed at RT due to fast relaxation and also not at LT (5 K) since these levels will not be occupied based on the Boltzmann distribution. A spin-vibrational coupling model for a better description of the system is needed to describe it more accurately, as was shown in Ref. 3.

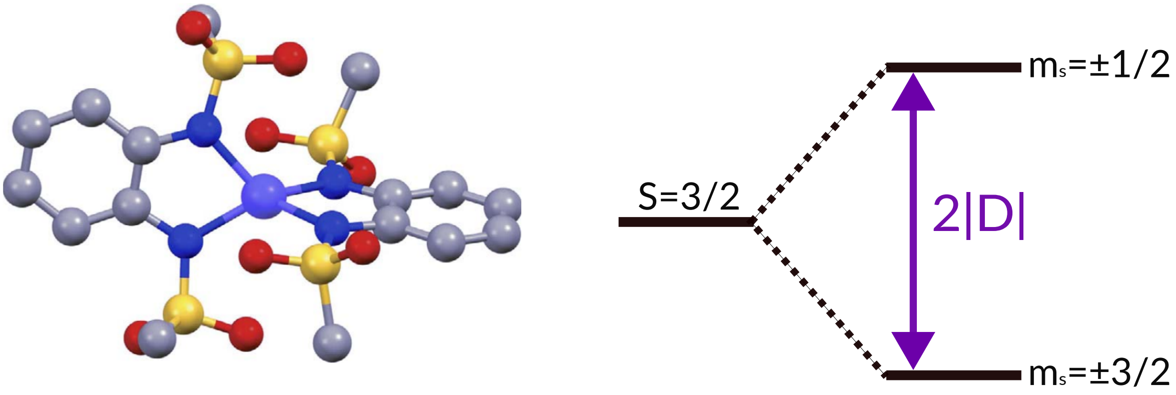

(a) (b)

Figure S12: The cobalt based SMM. **(a)** Crystallographic structure of a cobalt-based SMM. The cobalt is shown in blue, oxygen in red, sulfur in yellow, nitrogen in violet, and carbon in gray. The full chemical formula of the tetrahedral complex is $(HNEt_3)_2[Co^{II}(L^{2-})_2]$ where the ligand L stands for 1,2-bis(methanesulfonamido)benzene [3]. **(b)** The cobalt-based SMM Kramers system (non-integer spin number) $S = 3/2$ manifold. D is the axial ZFS parameter.

S10 XPS analysis

XPS measurements were carried out with Kratos AXIS ULTRA system using a monochromatic Al Kα X-ray source ($h\nu = 1486.6$ eV) at 75 W and detection pass energies ranging between 40 and 80 eV. Low-energy electron flood gun (eFG) was applied for charge neutralization. To define binding energies (BE) of different elements C 1s line at 284.8 eV [11, 12] was taken as a reference. Curve fitting analysis was based on linear or Shirley background subtraction and application of Gaussian-Lorentzian line shapes [13]. In Fig. S13a, one can observe a high resolution spectra in the cobalt 2p energy region before and after surface treatment (acid-cleaning). After drop-casting the material, and before acid cleaning, a clear signal of the cobalt is observed. On the other hand, there is no evidence for cobalt after acid cleaning the diamond. In Fig. S13b, we show the deconvoluted Co 2p signal (based on Ref. 14) after drop casting (and before acid cleaning the diamond). The peak maximum energy of the cobalt $2p_{3/2}$ main component is found at about 781 eV, and a second peak of the cobalt $2p_{1/2}$ is found at 796 eV. These peaks fit the Co^{2+} oxidation state of the element (we corrected for the binding energies due to utilizing a charge neutralizer). The shape of the Co 2p spectrum (main peaks with satellites) points upon the paramagnetic behavior of the cobalt.

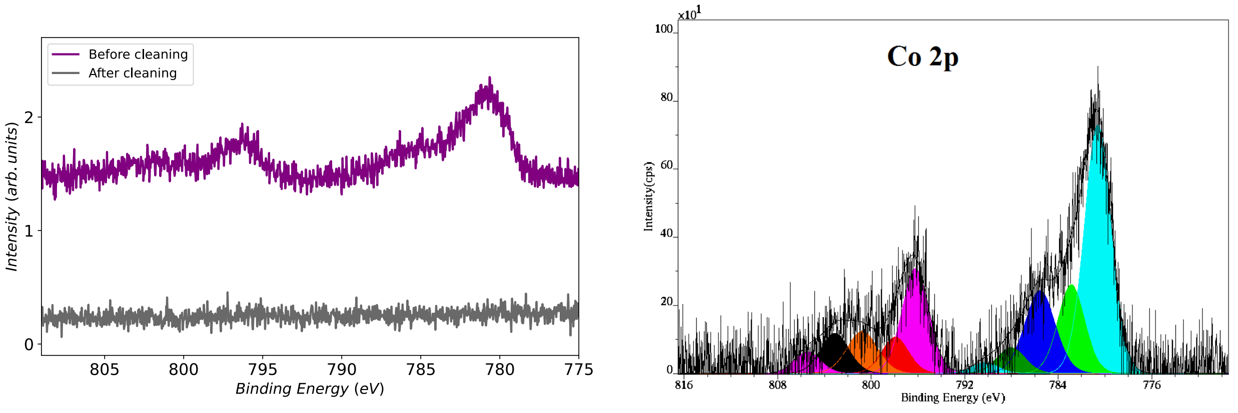

(a) (b)

Figure S13: XPS analysis for the diamond surface. (a) High-resolution Co 2p XPS spectra, before (purple) and after (gray) acid cleaning the diamond membrane. For clarity, the spectra were shifted on the intensity axis. (b) Deconvoluted Co 2p XPS spectrum [14], before acid cleaning the diamond membrane (purple curve in (a)).

S11 Experimental setup

For the experiments, both room temperature (RT) and low temperature (LT) optically integrated setups were based on confocal microscopy for NV center polarization and readout. A 520 nm green laser diode (DLnsec, LABS electronics), filtered (Thorlabs FBH520-10) and coupled into a single-mode fiber, provides optical excitation. The beam is directed onto the sample via a dichroic beam splitter (Thorlabs DMSP567) and focused with a semi-apochromat 100× objective (Olympus MPLFLN, NA = 0.9) at RT or an apochromatic objective (attocube LT-APO/VISIR, NA = 0.82) at LT. Scanning is performed with a Galvo scanner (or piezo stage in RT). Emitted red fluorescence is collected through the same path, filtered (Semrock FF01-731/137-25), and detected by two APDs in a *Hanbury-Brown and Twiss* configuration for photon correlation. A DC magnetic field is applied via a movable permanent magnet (RT) or superconducting coils (LT) using the system's three coils with a polar angle of 54.7° (w.r.t. the surface) and relative azimuthal angle of 120° one relative to each other. The LT system reaches 5 K under UHV conditions ($3 \cdot 10^{-9}$ mbar).

S12 ab-initio calculations

Density-functional calculations revealed that the Co site exhibits different magnetic moments between an isolated molecule and the molecule crystal. In a single-molecule, there are 5 electrons occupied for Co d orbitals with 3 spin down and 2 spin up, leading to a magnetic moment of $1\,\mu_B$. After forming a molecule crystal, the chemical environment changes, including bond lengths, bond angles and charge transfer, induces d^7 occupation with 5 spin down and 2 spin up, resulting in $3\,\mu_B$ magnetic moment (see Fig. S14).

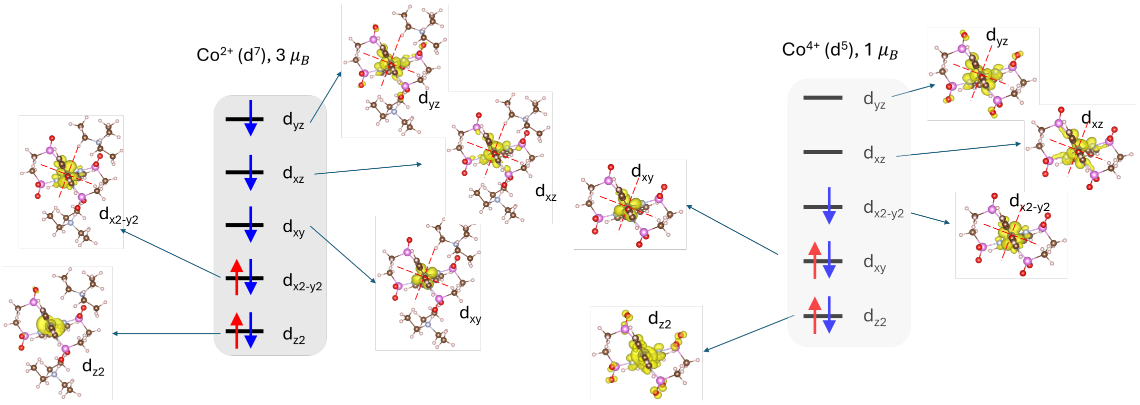

Figure S14: Left: Crystal field for the molecule in crystal. Right: Crystal field for an isolated molecule.

In an isolated molecule, the Co atom is located at the center of a heavily distorted tetrahedron formed by four N atoms (Fig. S15). The four Co–N bond lengths are approximately 1.87 Å. The six N–Co–N bond angles are divided into two groups: four angles range from 120.1° to 130.6°, and the other two are around 82.1°. As a result, the crystal field of the tetrahedron (e: d_{z2}, d_{x2-y2}; t_2: d_{xy}, d_{xz}, d_{yz}) undergoes further splitting. By analyzing the charge distribution of the energy levels in the molecule, we find that the Co d orbitals split into $\{d_{z2}, d_{xy}, d_{x2-y2}, d_{xz}, d_{yz}\}$ in ascending order of energy. Here, we have defined the cross line of the two Co–N–C planes as the z-axis. Notably, the d_{xy} orbital of the t_2 set exchanges its energy position with the d_{x2-y2} orbital of the e set in the molecule. The total magnetic moment of the isolated molecule is $1\,\mu_B$, arising from three spin-majority electrons in the three lowest Co orbitals and two spin-minority electrons in the two lowest Co orbitals.

When four molecules form a crystal with eight N–C–H clusters via hydrogen bonds (two for each individual molecule), the structure of the distorted N-tetrahedron undergoes significant changes. Specifically, the Co–N bond lengths increase to 2.00 Å, and the two groups of N–Co–N bond angles adjust to ranges of 121.2°–128.2° and approximately 81.6°, respectively. Additionally, the N–S bond length decreases from 1.7 Å in the isolated molecule to 1.6 Å in the crystal. These structural changes result in substantial alterations to the electronic structure. The total magnetic moment of the molecular crystal is $12\,\mu_B$, corresponding to $3\,\mu_B$ per individual molecule, with significantly enhanced spin polarization. In the crystal,

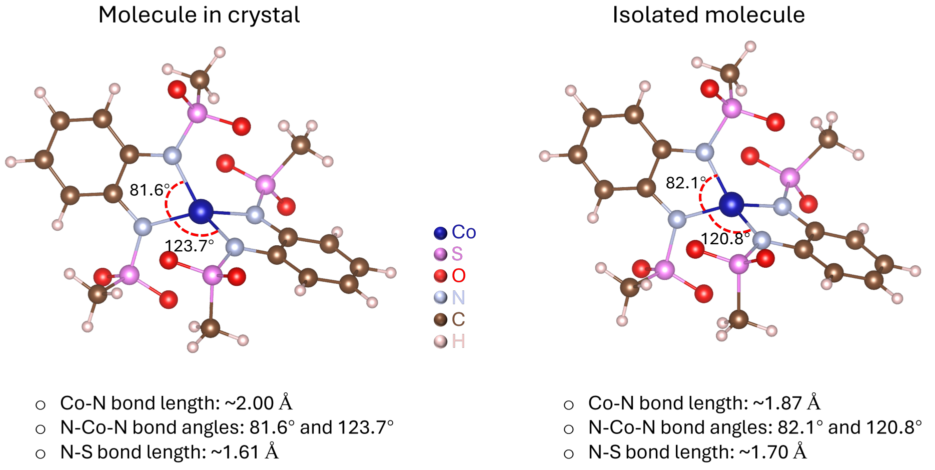

Figure S15: Structure comparison between the isolated molecule and the molecule in crystal

the Co d orbitals split into $\{d_{z2}, d_{x2-y2}, d_{xy}, d_{xz}, d_{yz}\}$ in ascending order of energy, using the same Cartesian coordinate system as the isolated molecule. Notably, the positions of the d_{xy} and d_{x2-y2} orbitals are exchanged compared to those in the isolated molecule. Intriguingly, each Co atom has five electrons in the spin-majority channel and two electrons in the spin-minority channel, resulting in a net magnetic moment of three Bohr magnetons. It is worth mentioning that the calculated magnetic moment of the molecular crystal aligns well with previous experimental results [3].

Compared to the isolated molecule, the bonding between the molecule and N–C–H clusters in the crystal alters the chemical valence of Co by donating two additional electrons to each Co atom. Spin-resolved charge transfer analysis indicates that these two electrons originate from the surrounding non-spin-polarized N and S atoms. In other words, the formation of the crystal not only transfers two electrons to Co but also fully spin polarizes them. To further validate this scenario, we introduced two additional electrons into the isolated molecule by assuming a homogeneous background charge. Accounting for the structural changes induced by charge doping, we found that the extra electrons become fully spin-polarized, occupying two d orbitals in the spin-majority channel of Co. This results in a net magnetic moment of 3 μ_B, closely resembling that of the molecular crystal. Moreover, while the energy differences between adjacent d orbitals vary, their overall sequence remains consistent with that observed in the crystal. These findings unambiguously demonstrate that the N–C–H organic ligands significantly alter the molecule's properties in the crystal by introducing additional charges — an effect that should be experimentally detectable.

Methods

All calculations were performed using density functional theory (DFT) as implemented in VASP [15]. The generalized gradient approximation (GGA) was employed for the exchange-

correlation interactions [16]. Structures were fully relaxed until the residual force on each atom was below 10 meV/Å, utilizing an energy cutoff of 520 eV for the plane-wave basis set. To model the isolated molecule, a periodic lattice identical to that of the molecular crystal was employed. This lattice was sufficiently large to prevent interactions between periodic images of the molecule. All calculations sampled the Brillouin zone using only the Γ point.

S13 The T_1 protocol

For performing the T_1 longitudinal relaxometry experiments, we used the following pulse sequence while using no MW radiation (due to a technical difficulty in our setup when using microwave with long pulse sequences).

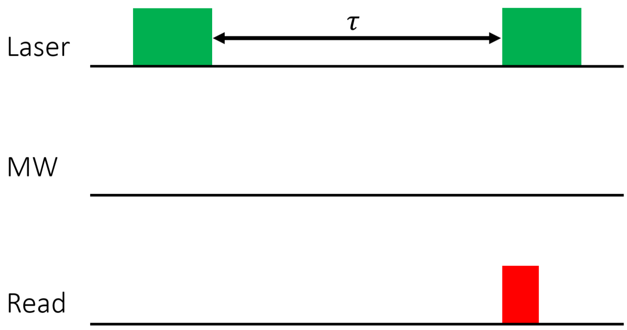

Figure S16: The T_1 pulse sequence. The first laser pulse is used for initializing the NV center to the $|0\rangle$ state and the second laser pulse is used for readout and initialization for the next repetition of the sequence. In our experiments, the interpulse delay τ ranges from microseconds to milliseconds.

We note that while in some samples operating in ultra-high vacuum conditions, shallow NVs can experience charge-state instability [17], in the two diamond samples used in this work, such "quenching" events (the switching from the negatively-charged state, NV^- to the neutral-charge state, NV^0) were not observed. We also note that in other experiments performed in the same system, we indeed sometimes observe this charge-state instability when working with shallow NVs.

S14 Quantum sensing with NV centers

The Hamiltonian of the ground state of the NV spin system, under some simplifying assumptions, excluding the interaction with nearby spins, is

$$\hat{\mathcal{H}} = D\hat{S}_z^2 + \gamma_{NV}\left(B_0 + \Delta B_z(t)\right)\hat{S}_z + \gamma_{NV}B_1(t)\hat{S}_x$$

where we set the natural axis of the NV to be $\hat{z}$. $D = 2.87$ GHz is the zero-field splitting (ZFS) and $\gamma_e = 28$ GHz/T is the gyromagnetic ratio of the NV electronic spin. The second term describes the interaction between an applied magnetic field and the NV electron spin. A DC magnetic field B_0, which is aligned with the quantization axis of the NV, removes the system's degeneracy and results in three separated levels. An AC varying magnetic field $\Delta B_z(t)$ can be sensed in the superposition state due to phase accumulation. Say with the $|m_s = 0\rangle$,$|m_s = +1\rangle$ states we have:

$$|\psi(t)\rangle = \frac{1}{\sqrt{2}}\left(|0\rangle + e^{i\varphi(t)}\,|1\rangle\right)$$

where the phase is essentially

$$\varphi(\tau) = \varphi(0) + \int_o^\tau \hat{\mathcal{H}}_{Zeeman}(t)dt = \varphi(0) + \gamma B_0\tau + \gamma\int_o^\tau \Delta B_z(t)dt \qquad \text{(S5)}$$

The third term of the Hamiltonian describes the interaction with a transverse magnetic field $B_1(t)$ set along the $\hat{x}$ direction. Through this interaction, the system can be transferred into the superposition state by applying a $\pi/2$ pulse with the correct Larmor frequency. Essentially, in this way, manipulations of the spin can be carried out, and various designated pulse sequences can be utilized. However, through this term, the system is also influenced by random AC magnetic fields along the transverse plane, such that it will eventually reach thermal equilibrium (the maximally mixed state in our case). This relaxation, which is mainly phonon-dominated, limits the possible sensing time with NVs and is known as the longitudinal relaxation time T_1 [18]. Another timescale that characterizes the system is the transverse relaxation time or the coherence time T_2 of the superposition state. The T_2 time is limited by the surrounding noise bath, which can randomize the accumulated phase (Eq. S5).

References

[1] Flinn, B. T. *et al.* Nitrogen vacancy defects in single-particle nanodiamonds sense paramagnetic transition metal spin noise from nanoparticles on a transmission electron microscopy grid. *Nanoscale Advances* **5**, 6423–6434 (2023).

[2] Steinert, S. *et al.* Magnetic spin imaging under ambient conditions with sub-cellular resolution. *Nat. Commun.* **4**, 1607 (2013).

[3] Rechkemmer, Y. *et al.* A four-coordinate cobalt(II) single-ion magnet with coercivity and a very high energy barrier. *Nat. Commun.* **7**, 10467 (2016).

[4] Kumar, J. *et al.* Room temperature relaxometry of single nitrogen vacancy centers in proximity to α-$RuCl_3$ nanoflakes. *Nano Lett.* **24**, 4793–4800 (2024).

[5] Jarmola, A., Acosta, V. M., Jensen, K., Chemerisov, S. & Budker, D. Temperature- and magnetic-field-dependent longitudinal spin relaxation in nitrogen-vacancy ensembles in diamond. *Phys. Rev. Lett.* **108**, 197601 (2012).

[6] Davis, E. J. *et al.* Probing many-body dynamics in a two-dimensional dipolar spin ensemble. *Nat. Phys.* **19**, 836–844 (2023).

[7] Slichter, C. P. *Principles of magnetic resonance* Vol. 1 (Springer Science & Business Media, 2013).

[8] de Sousa, R. *Electron Spin as a Spectrometer of Nuclear-Spin Noise and Other Fluctuations*, 183–220 (Springer Berlin Heidelberg, 2009).

[9] Ziegler, J. F. & Biersack, J. P. *The Stopping and Range of Ions in Matter*, 93–129 (Springer US, 1985).

[10] Jelezko, F. & Wrachtrup, J. Single defect centres in diamond: A review. *physica status solidi (a)* **203**, 3207 (2006).

[11] G. Beamson, D. B. High resolution XPS of organic polymers, the Scienta ESCA300 database. *Adv. Mater.* **5**, 778–778 (1993).

[12] Moulder, J. F., Stickle, W. F., Sobol, P., Bomben, K. & Chastain, J. Handbook of X-ray Photoemission Spectroscopy. *Perkin-Elmer Corporation, Minnesota* (1992).

[13] Shirley, D. A. High-resolution X-ray photoemission spectrum of the valence bands of gold. *Phys. Rev. B* **5**, 4709–4714 (1972).

[14] Biesinger, M. C. *et al.* Resolving surface chemical states in xps analysis of first row transition metals, oxides and hydroxides: Cr, Mn, Fe, Co and Ni. *Applied Surface Science* **257**, 2717–2730 (2011-01).

[15] Kresse, G. & Furthmüller, J. Efficient iterative schemes for ab initio total-energy calculations using a plane-wave basis set. *Phys. Rev. B* **54**, 11169–11186 (1996).

[16] Perdew, J. P., Burke, K. & Ernzerhof, M. Generalized gradient approximation made simple. *Phys. Rev. Lett.* **77**, 3865–3868 (1996).

[17] Neethirajan, J. N. *et al.* Controlled surface modification to revive shallow NV^- centers. *Nano Lett.* **23**, 2563–2569 (2023).

[18] Bandyopadhyay, S. & Cahay, M. *Introduction to Spintronics, Second Edition* (Taylor & Francis, 2015).
